# BALB/c Mice Infected with Antimony Treatment Refractory Isolate of *Leishmania braziliensis* Present Severe Lesions due to IL-4 Production

**DOI:** 10.1371/journal.pntd.0000965

**Published:** 2011-03-01

**Authors:** Diego L. Costa, Vanessa Carregaro, Djalma S. Lima-Júnior, Neide M. Silva, Cristiane M. Milanezi, Cristina R. Cardoso, Ângela Giudice, Amélia R. de Jesus, Edgar M. Carvalho, Roque P. Almeida, João S. Silva

**Affiliations:** 1 Department of Biochemistry and Immunology, Ribeirão Preto Medical School, University of São Paulo, Ribeirão Preto, Brazil; 2 Biomedical Sciences Institute, Federal University of Uberlândia, Uberlândia, Brazil; 3 Department of Biological Sciences, Federal University of Triângulo Mineiro, Uberaba, Brazil; 4 Immunology Service, Professor Edgar Santos Universitary Hospital, Federal University of Bahia, Salvador, Brazil; 5 Department of Internal Medicine and Pathology, Federal University of Sergipe, Aracajú, Brazil; Institut Pasteur de Tunis, Tunisia

## Abstract

**Background:**

*Leishmania braziliensis* is the main causative agent of cutaneous leishmaniasis in Brazil. Protection against infection is related to development of Th1 responses, but the mechanisms that mediate susceptibility are still poorly understood. Murine models have been the most important tools in understanding the immunopathogenesis of *L. major* infection and have shown that Th2 responses favor parasite survival. In contrast, *L. braziliensis*–infected mice develop strong Th1 responses and easily resolve the infection, thus making the study of factors affecting susceptibility to this parasite difficult.

**Methodology/Principal Findings:**

Here, we describe an experimental model for the evaluation of the mechanisms mediating susceptibility to *L. braziliensis* infection. BALB/c mice were inoculated with stationary phase promastigotes of *L. braziliensis*, isolates LTCP393(R) and LTCP15171(S), which are resistant and susceptible to antimony and nitric oxide (NO), respectively. Mice inoculated with LTCP393(R) presented larger lesions that healed more slowly and contained higher parasite loads than lesions caused by LTCP15171(S). Inflammatory infiltrates in the lesions and production of IFN-γ, TNF-α, IL-10 and TGF-β were similar in mice inoculated with either isolate, indicating that these factors did not contribute to the different disease manifestations observed. In contrast, IL-4 production was strongly increased in LTCP393(R)-inoculated animals and also arginase I (Arg I) expression. Moreover, anti-IL-4 monoclonal antibody (mAb) treatment resulted in decreased lesion thickness and parasite burden in animals inoculated with LTCP393(R), but not in those inoculated with LTCP15171(S).

**Conclusion/Significance:**

We conclude that the ability of *L. braziliensis* isolates to induce Th2 responses affects the susceptibility to infection with these isolates and contributes to the increased virulence and severity of disease associated with them. Since these data reflect what happens in human infection, this model could be useful to study the pathogenesis of the *L. braziliensis* infection, as well as to design new strategies of therapeutic intervention.

## Author Summary

Leishmaniasis is a neglected disease that affects more than 12 million people worldwide. In Brazil, the cutaneous disease is more prevalent with about 28,000 new cases reported each year, and *L. braziliensis* is the main causative agent. The interesting data about the infection with this parasite is the wide variety of clinical manifestations that ranges from single ulcerated lesions to mucocutaneous and disseminated disease. However, experimental models to study the infection with this parasite are difficult to develop due to high resistance of most mouse strains to the infection, and the mechanisms underlying the distinct manifestations remain poorly understood. Here, the authors use a mouse experimental model of infection with different *L. braziliensis* isolates, known to induce diseases with distinct severity in the human hosts, to elucidate immune mechanisms that may be involved in the different manifestations. They showed that distinct parasite isolates may modulate host response, and increased IL-4 production and Arg I expression was related to more severe disease, resulting in longer length of disease with larger lesions and reduced parasite clearance. These findings may be useful in the identification of immunological targets to control *L. braziliensis* infection and potential clinical markers of disease progression.

## Introduction

Leishmaniasis comprises several diseases caused by protozoans of the genus *Leishmania*. The most common disease in Brazil is American tegumentary leishmaniasis (ATL), caused by *L. braziliensis*. In the Americas, the main invertebrate vectors for this parasite, are sand flies of the genus *Lutzomyia* [1]. The most studied parasite of the genus *Leishmania* is *L. major*. Human infection with this pathogen is generally benign and eventually resolves spontaneously, resulting in lifelong immunity. On the other hand, infection with *L. braziliensis* is chronic and causes latency, which may lead to parasite dissemination to the nasal and oral mucosa years after resolution. Even chemotherapeutic treatment does not exclude the possibility of developing mucocutaneous leishmaniasis [2]–[4].

Protection against, and susceptibility to *L. major*, another cutaneous leishmaniasis-causing parasite, have been clearly established in mouse models of infection. In mice, Th1 responses [5] involving nitric oxide (NO), and the cytokines IL-12, IFN-γ and TNF-α [6], [7], result in parasite killing [8], [9]. In contrast, Th2 responses, which are characterized by the production of IL-4, IL-13 and IL-10, result in susceptibility to infection [10]-[13]. CD4^+^CD25^+^ regulatory T cells are also important sources of IL-10 and contribute significantly to susceptibility [14], [15]. Similarly, Th1 responses have been shown to be necessary for parasite killing in mouse models of *L. braziliensis* infection [16]; however, it has been difficult to develop experimental models for studying susceptibility factors because most mouse strain develop strong Th1 responses that easily control *L. braziliensis* infection [17]. In humans, cutaneous (CL) and mucocutaneous leishmaniasis (ML) caused by *L. braziliensis* infection are also associated with a strong production of Th1 cytokines and marked migration of inflammatory mononuclear cells to lesion sites [18]–[22]; although spontaneous resolution is observed in only 30% of patients [23]. Also a large range of clinical manifestations is observed in patients with *L. braziliensis* cutaneous leishmaniasis, showing a great difference between the diseases caused by *L. major* and *L. braziliensis*. A complex interplay between host traits and intrinsic properties of the parasite *L. braziliensis* that contribute to the variety of clinical presentations is not clearly understood. Experimental infection of mice with two *L. braziliensis* isolates from patients with either mild or severe lesions resulted in distinct clinical features and different patterns of chemokine production; however, no differences were observed in parasite replication [24], [25]. Intrinsic characteristics of the parasites that result in increased capacity to survive inside human macrophages, such as resistance to nitric oxide (NO) in some *L. braziliensis* and *L. amazonensis* isolates, have been associated with more severe forms of the disease [26]. Despite these findings, the true immunological mechanisms that mediate susceptibility to *L. braziliensis* remain poorly understood.

To address this issue, we developed an experimental model in which BALB/c mice were inoculated with stationary phase promastigotes from *L. braziliensis* isolates obtained from CL patients that were refractory or responsive to antimony treatment, and that presented different severities of disease manifestations. We characterized the experimental infection with these two isolates in mice and found that similar to the difference in disease severity between the human hosts from which these strains were isolated, the resistant isolate caused a more severe disease in mice than the susceptible isolate. The increased lesion development caused by increased parasitic replication was associated with the production of IL-4 in response to the resistant isolate. This interesting model of infection can be useful to further studies to understand the variability of clinical manifestations of the disease and to design immunological targets to be used to control the infections.

## Materials and Methods

### Mice and parasites

Female 6–8 week old BALB/c mice were used in all experiments. The animals were maintained at the animal holding facility of the department of Biochemistry and Immunology of the Ribeirão Preto Medical School – University of São Paulo, and all procedures were approved by the local ethics committee for animal care and research “Ethics Committee in Animal Research of the FMRP-USP”. *Leishmania braziliensis* isolates LTCP393(R) and LTCP15171(S) were obtained from cutaneous ulcers of patients from the endemic area of Corte de Pedra, BA, Brazil, specifically for this study, and all human subjects were briefed on procedures and signed informed consent documentation. All work with human subjects was carried out under “Federal University of Bahia Ethical Committee” approval number 5/2006. *L. braziliensis* LTCP15171(S) was isolated from a 38-year-old male patient who had a single skin ulcer with an area of 120 mm^2^ that was cured after one course of antimony therapy. *L. braziliensis* LTCP393(R) was isolated from a 26-year-old male patient that had several skin lesions ranging in size from 100 to 1575 mm^2^ and mucosal disease. This patient was treated with several courses of antimony, pentoxifylin, and amphotericin B over a period of 20 years before being cured. These parasites were isolated in N.N.N. media and frozen in liquid nitrogen immediately after isolation.

### Parasite Characterization

Promastigotes were grown in Schneider's insect medium (Sigma, Saint Louis, USA) supplemented with 20% heat-inactivated fetal calf serum (Cultilab, Campinas, SP, Brazil), 4 mM NaHCO_3_, 100 U/ml penicillin, 100 µg/ml streptomycin (all from Gibco, Grand Island, NY, USA), and 2% v/v male human urine at 25°C. At 5^th^ day of culture, both isolates reached stationary phase, after inoculation of 1×10^6^ promastigotes in 6 ml of medium. The amount of metacyclic parasites at the chosen day was analyzed using Ficoll 400 (Pharmacia-GE Healthcare, Uppsala, Sweden) gradient as described [27], [28], and was similar for LTCP393(R) and LTCP15171(S) isolates (0.8050%±0.01528 and 1.132%±0.2122, respectively). NO resistance assays were performed as described [26], [29]. LTCP393(R) was resistant to 16 mM NaNO_2_ (NO donor), and LTCP15171(S) was susceptible to 4 mM NaNO_2_. Antimony resistance was tested by culturing BALB/c mice peritoneal macrophages isolated and cultured as described [30], in the presence or absence of 10, 30 and 90 µg/ml Glucantime (Sanofi Aventis Farmacêutica, Suzano,SP, Brazil), for 24, 48 and 72 h. Compared to non-treated infected cells, only in LTCP15171(S)-infected macrophages there was a substantial reduction in intracellular parasite numbers. We therefore will refer to these parasites as resistant to antimony and NO (LTCP393(R)), and susceptible to antimony and NO (LTCP15171(S)). To determine the infectivity of the isolates, macrophages were incubated with 5 and 10 parasites per cell and the percentage of infected cell and number of intracellular parasites determined. Before the beginning of the experiments, the isolates were submitted to passages into hamsters and BALB/c mice, for recovering of virulence and selection of parasites. During the experiments, parasite virulence was assured by constant maintenance and re-isolation from infected BALB/c mice.

### Parasite inoculation, lesion measurement and parasite load estimate

We used a well established protocol of inoculation [31]–[35], in which the right ear dermis of BALB/c mice was inoculated with stationary phase promastigotes (10^6^ parasites in 10 µl of sterile saline) using a 27.5-gauge needle. Lesion size, which was defined as the difference in thickness between the infected ear and the non-infected contralateral ear, was monitored weekly using a digital caliper (Mitutoyo, Suzano, SP, Brazil). The parasite load was determined using a quantitative limiting dilution assay as previously described [36].

### Antibody treatment

The rat anti-IL-4 mAb was purified from ascites of mice injected with the hybridoma 11B11. IL-4 was neutralized by intraperitoneal (i.p.) injection of 2 mg purified mAb one day before infection. Additional i.p. injections of 1 mg purified mAb were performed twice a week for seven weeks. Controls received 1 mg of normal rat IgG diluted in PBS.

### Cell isolation from lesions and lymph nodes

Ears from infected mice were collected and incubated at 37°C for one hour in RPMI-1640 medium containing 2 mM L-glutamine, 100 U/ml penicillin, 100 µg/ml streptomycin (all from Gibco) and 500 µg/ml Liberase CI (Roche, Basel, Switzerland). The tissues were processed inside Medcons using a Medimachine (both from Becton & Dickinson Biosciences, San Diego, CA, USA). After processing, the cells were filtered through a 50 µm filter, viability was assessed by trypan blue exclusion, and the cell concentration was adjusted.

### Flow cytometry

Immunostaining was performed with anti-CD3, CD19, CD4, CD8, CD25, CD11b, CD11c and Gr1 antibodies conjugated to FITC, PE or PerCP fluorochromes. For regulatory T cell phenotyping, CD4^+^CD25^+^ cells were stained with anti-FoxP3, CD103, CTLA-4 and GITR antibodies conjugated to PE. For intracellular staining, the cells were permeabilized with a Cytofix/Cytoperm kit (BD Biosciences) according to the manufacturer's guide. For all analyses, the results were compared to those obtained with cells stained with isotype control antibodies (all antibodies were from BD Biosciences and eBiosciences, San Diego, CA, USA). Cell acquisition was performed using a FACSort flow cytometer and CellQuest software (BD Biosciences). Data were plotted and analyzed using Cell Quest (BD Biosciences) and FlowJo software (Tree Star, Ashland, OR).

### Cell cultures

Single cell suspensions of draining retromaxilar lymph nodes were prepared aseptically, diluted to a concentration of 2×10^6^ cells/ml and dispensed into 48-well plates in a total volume of 500 µl of complete RPMI-1640 medium (Gibco) (1×10^6^ cells/well) with or without live stationary phase *L. braziliensis* promastigotes at a ratio of five parasites to one cell (5×10^6^ parasites/well). Cell culture supernatants were harvested after 72 h of culture at 37°C in 5% CO_2_, and the levels of IFN-γ, IL-4, IL-10 and TGF-β were determined by ELISA using commercial kits (BD Biosciences and R&D Systems, Minneapolis, MN, USA).

### Real time PCR

Total RNA was extracted from whole fragments of infected ears using the Promega RNA extraction kit (Promega, Madison, WI, USA). RNA was quantified using a spectrophotometer (BioMate 3; Thermo Spectronic, Rochester, NY, USA) and cDNA was synthesized using 1 µg RNA through an RT reaction using ImProm-II reagents (Promega) according to the manufacturer's instructions. Real-time PCR quantitative mRNA analyses were performed using the Platinum SYBR Green qPCR SuperMix-UDG with ROX reagents (Invitrogen, Carlsbad, CA, USA) and the ABI Prism 7000 sequence detection system (Applied Biosystems, Warrington, UK). The sequences of murine primers were designed using PrimerExpress software (Applied Biosystems) and nucleotide sequences present in the GenBank database. Relative gene expression levels were calculated according to the instructions from the Applied Biosystems user's bulletin (P/N 4303859). Briefly, gene expression levels were normalized to the level of β-actin in each sample using the cycle threshold (Ct) method. Negative controls without RNA and without RT were also performed. The threshold for positivity of real-time PCR was determined based on the expression of the specific mRNAs in the ears of uninfected mice using the equation 2^−ΔΔCt^. Murine primer sequences utilized: β-actin sense: 5′- AGC TGC GTT TTA CAC CCT TT - 3′, β actin anti-sense: 5′ - AAG CCA TGC CAA TGT TGT CT – 3′; IFN-γ sense: 5′- GCA TCT TGG CTT TGC AGC T – 3′, IFN-γ anti-sense: 5′ - CCT TTT TCG CCT TGC TGT TG – 3′; IL-4 sense: 5′ - GAA TGT ACC AGG AGC CAT ATC – 3′, IL-4 anti-sense: 5′ - CTC AGT ACT ACG AGT AAT CCA – 3′; IL-10 sense: 5′ - TGG ACA ACA TAC TGC TAA CCG – 3′, IL-10 anti-sense: 5′ - GGA TCA TTT CCG ATA AGG CT – 3′; TGF-β sense: 5′ - GCT GAA CCA AGG AGA CGG AAT – 3′, TGF-β anti-sense: 5′ - GCT GAT CCC GTT GAT TTC CA – 3′; TNF-α sense: 5′ - TGT GCT CAG AGC TTT CAA CAA - 3′, TNF-α anti-sense: 5′ - CTT GAT GGT GGT GCA TGA GA – 3′; inducible nitric oxide synthase (iNOS) sense: 5′ - CGA AAC GCT YCA CTT CCA A – 3′, iNOS anti-sense: 5′ - TGA GCC TAT ATT GCT GTG GCT – 3′; arginase I (Arg I) sense: 5′ - GTT CCC AGA TGT ACC AGG ATT C – 3′, Arg I anti-sense: 5′ - CGA TGT CTT TGG CAG ATA TGC – 3′. Murine Arg I primer was tested for cross-hybridization with the parasite ortholog, and no cross-hybridization or amplification occurred in the parasite material.

### Statistical analyses

Data were expressed as means ± standard errors of the means (SEM). Student's *t* test and ANOVA followed by Tukey's multiple comparison test were used to analyze the statistical significance of the observed differences between the mice infected with LTCP393(R) or LTCP15171(S) when samples had a normal distribution, and Mann-Whitney tests were used when the distribution was not normal. Differences were considered significant when *P<*0.05. All analyses were performed using Prism software version 5.0 (GraphPad San Diego, CA, USA).

## Results

### Pattern of experimental infection with LTCP393(R) and LTCP15171(S) in BALB/c mice

To examine the kinetics of lesion development, 5^th^ day of culture stationary phase parasites, with similar amount of metacyclics, were inoculated in the right ear dermis of BALB/c mice, and lesions were measured weekly using a caliper for 15 weeks following infection.

**Figure 1:**
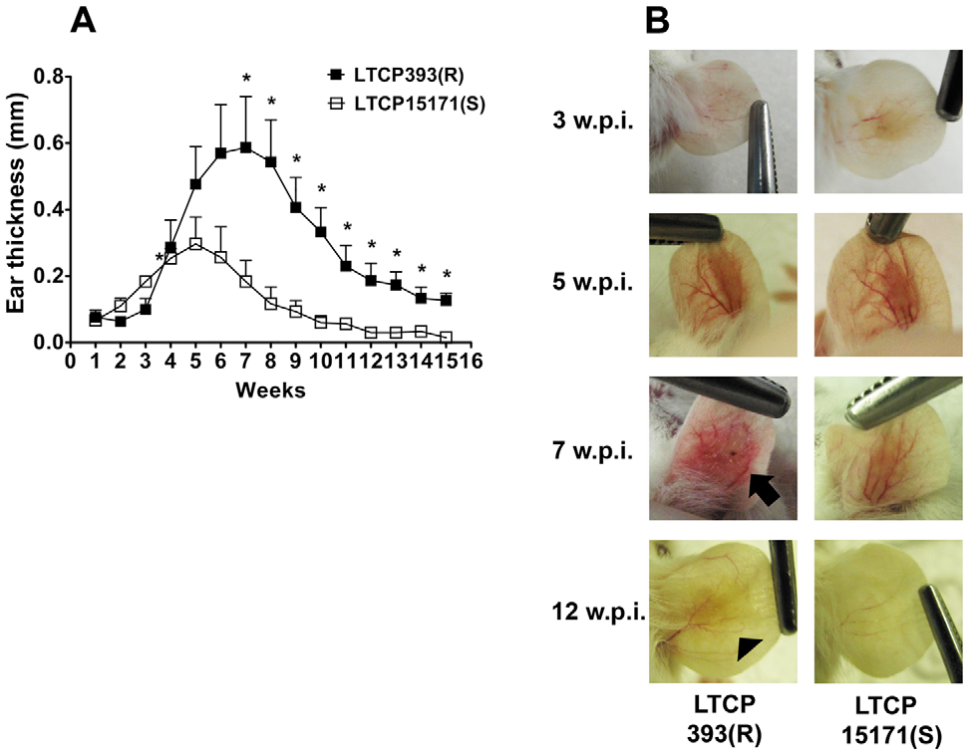
LTCP393(R)-inoculated BALB/c mice present larger lesions. (A) The animals were inoculated with 1×10^6^ *Leishmania braziliensis* stationary phase promastigotes, isolates LTCP393(R) (black squares) or LTCP15171(S) (white squares) in the right ear dermis and the course of lesion development was monitored for 15 weeks. Lesion thickness was determined as the difference between the infected ear and the contralateral one, noninfected. Results are expressed as mean ± standard error of mean, and are representative of 2–3 independent experiments. (B) Photographs of mouse ears at 3, 5, 7 and 12 weeks post infection (w.p.i.). The arrow indicates ulceration in the ear of an animal infected with LTCP393(R) isolate at 7 weeks post infection, and the arrowhead indicates remaining lesion at 12 weeks post infection in animals infected with LTCP393(R) isolate. *p<0,05.

**Figure 2:**
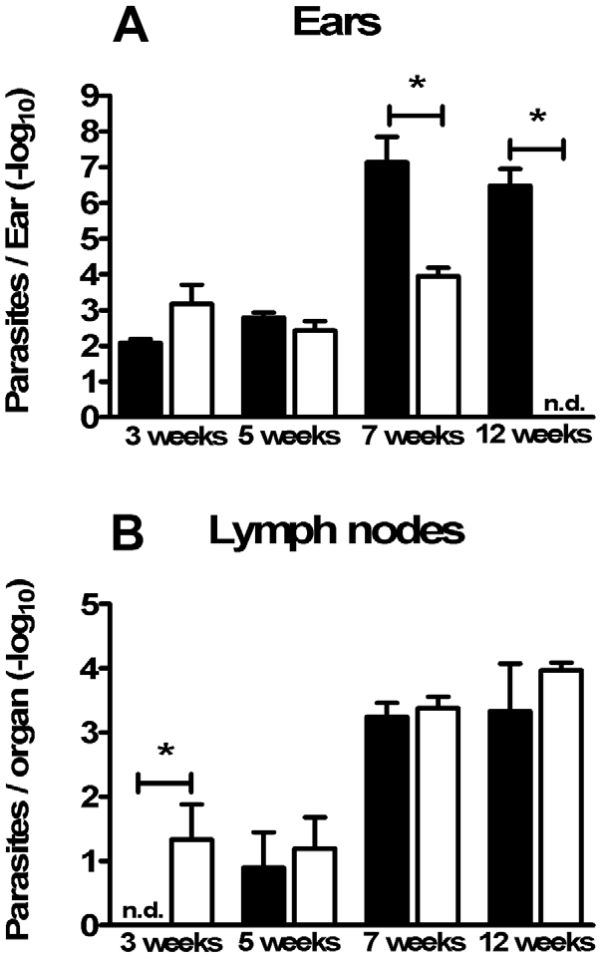
LTCP393(R)-inoculated BALB/c mice present higher parasite loads in the lesion sites. Parasite load estimate in ears (A) and lymph nodes (B) of BALB/c mice inoculated at right ear dermis with 1×10^6^ stationary phase promastigotes of *L. braziliensis*, isolates LTCP393(R) (black bars) or LTCP15171(S) (white bars), at 3, 5, 7 and 12 weeks post infection. Results are expressed as mean ± standard error of mean, and are representative of 3 independent experiments. n.d. = non-detected. *p<0,05.

Mice controlled the lesions caused by both isolates, but kinetics of lesion development was different between the groups and the severity of lesions was higher in LTCP393(R)-infected mice. Fifteen weeks after infection, the lesions in mice inoculated with LTCP15171(S) were completely healed, while small lesions still remained in mice inoculated with LTCP393(R) (Fig. 1A). On the third week after infection, the lesions in animals inoculated with LTCP15171(S) were approximately twice the size of those in mice inoculated with LTCP393(R) (Fig. 1A and 2B). Five weeks post-infection, the lesions in the animals inoculated with LTCP15171(S) reached their peak size and began to decrease in thickness, while the lesions in mice infected with LTCP393(R) continued to develop and reached their peak size seven weeks post-infection. At this time point, the lesions in mice inoculated with LTCP393(R) were approximately three times thicker than those in mice inoculated with LTCP15171(S) and two times thicker than the peak size of the lesions in mice inoculated with LTCP15171(S), which was reached five weeks post-infection (Fig. 1A). Also in this time point, the lesions were ulcerated in the mice inoculated with LTCP393(R) (Fig. 1B, arrow). Eight weeks post-infection, the lesions in mice inoculated with the LTCP393(R) isolate began to regress, but these lesions remained significantly larger than those in mice inoculated with LTCP15171(S), and small lesions were present in mice inoculated with LTCP393(R) after 12 weeks (Fig. 1B, arrowhead).

We next determined the infective capacity of the stationary phase promastigotes of both isolates, by quantifying the amount of amastigotes inside mouse peritoneal macrophages, after 6 h incubation. LTCP393(R) and LTCP15171(S) parasites infected 57.14%±9.787 and 58.38%±5.558 the cells, respectively. Moreover, the amount of intracellular parasites was similar (347.7±47.75 versus 384.7±20.30 amastigotes of the isolates LTCP393(R) and LTCP15171(S), respectively, per 100 macrophages, using 5 parasites/cell to the infection). Similar data was found using different ratio of parasites/cell. We concluded that both isolates infect macrophages with the same efficacy.

The number of parasites in the infected ears and in the draining lymph nodes at specific time points post-infection was determined using a previously described limiting dilution assay [36]. The parasite loads in the earlier phases of the experimental infection were analyzed (1, 3, 7 and 14 days post-infection) in ears and draining lymph nodes. Therefore, no difference was found between the groups, showing that *in vivo*, as observed previously *in vitro*, both parasite isolates presented the same infectivity rate with the chosen inocula. At time points three and five weeks post-infection, also no differences in parasite load were observed between the groups. Seven weeks post-infection, a significantly higher number of parasites was present in the ears of mice inoculated with LTCP393(R) than those of mice inoculated with LTCP15171(S) (2.01×10^8^±1.63×10^8^ and 1.73×10^4^±1.1×10^4^, respectively; p = 0.0079). Twelve weeks after infection, no parasites were detected in the ears of animals inoculated with the susceptible isolate, but the ears of mice inoculated with the resistant isolate contained an average of 1.79×10^7^±1.08×10^7^ parasites (Fig. 2A).

**Figure 3:**
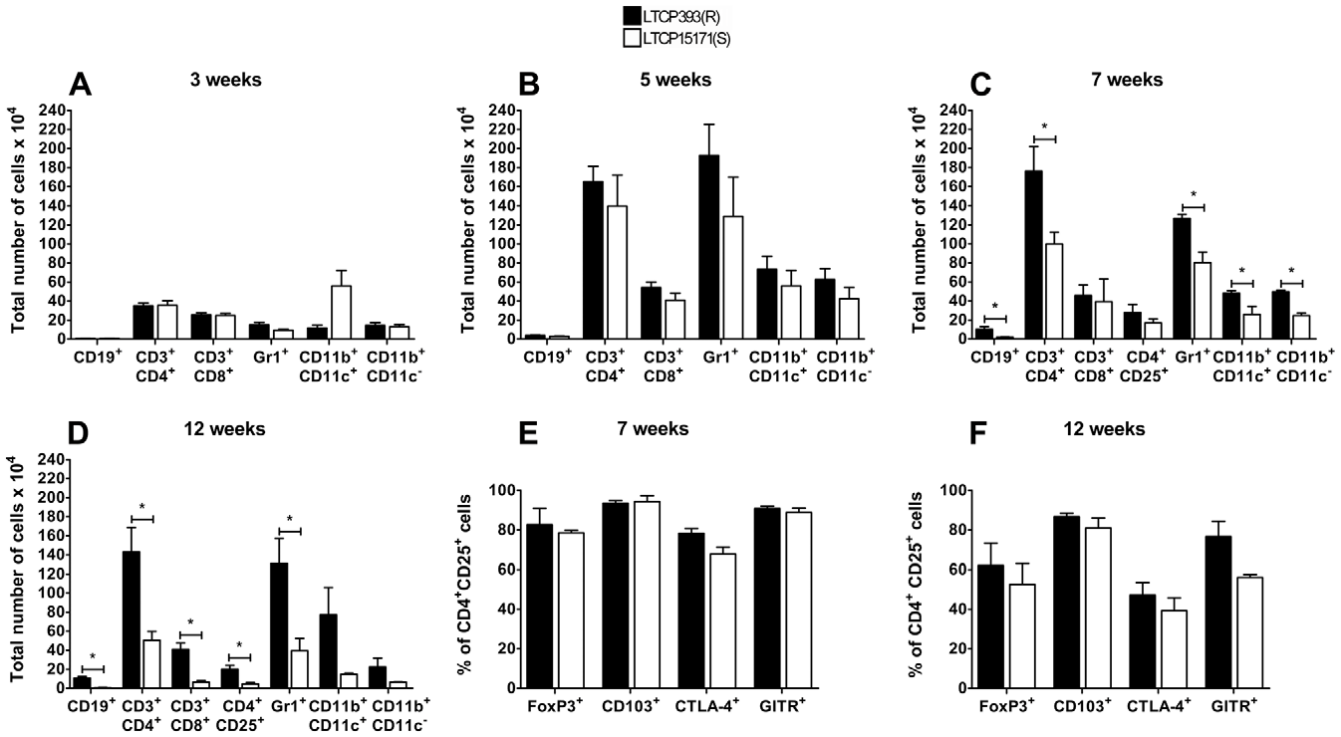
Flow cytometry of infected BALB/c mice ears inflammatory infiltrate. The animals were inoculated with 1×10^6^ stationary phase *L. braziliensis* promastigotes, isolates LTCP393(R) (black bars) or LTCP15171(S) (white bars). Analyses were conducted at 3, 5, 7 and 12 weeks post infection, FoxP3, CD103, CTLA-4 and GITR expression on CD4^+^CD25^+^ cells were evaluated at 7 and 12 weeks post infection. Results are expressed as total number of cells in the lesion (A, B, C and D), and as percentage of cells inside CD4^+^CD25^+^ gate (E and F). Results are expressed as mean ± standard error of mean, and are representative of 3 independent experiments. *p<0,05.

Fewer parasites were detected in the draining lymph nodes than in the ears of infected mice. Three weeks post-infection, the number of parasites detected in the lymph nodes of mice inoculated with LTCP15171(S) was significantly higher than that in lymph nodes of mice inoculated with LTCP393(R) (103.7±45.94 and 0, respectively). Five weeks after infection, no significant difference was observed between the number of parasites in lymph nodes from mice inoculated with LTCP393(R) (70.53±43.47) and that in lymph nodes from mice inoculated with LTCP15171(S) (61.32±27.55). Despite the regression of lesions in the ears at subsequent time points post-infection, the parasite loads in the lymph nodes increased significantly (LTCP393(R), p = 0.0112 and p = 0.0398 at seven and 12 weeks post-infection, respectively, compared to five weeks post-infection; LTCP15171(S), p = 0.0119 and p = 0.0080 at seven and 12 weeks post-infection, respectively, compared to five weeks post-infection); however, no significant differences in parasite loads were detected between mice inoculated with the resistant or susceptible isolates (Fig. 2B). We next characterized the inflammatory infiltrates present in the lesions of mice experimentally infected with each isolate using flow cytometry. There were no differences in the cell populations studied three weeks after infection (Fig. 3A). Five weeks after infection, the numbers of most cell types, and especially of the CD4^+^ T lymphocytes and neutrophils (GR-1^+^), increased significantly in infiltrates in mice infected with either isolate; however, CD8^+^ T lymphocytes, dendritic cells (CD11b^+^CD11c^+^) and macrophages (CD11b^+^CD11c^−^) were not significantly increased in infiltrates in LTCP15171(S)-inoculated mice. No differences in the cell populations were detected between mice infected with resistant or susceptible isolates at this time point (Fig. 3B).

Seven weeks after infection, more B lymphocytes were present in lesions of animals inoculated with LTCP393(R) than in those of animals inoculated with LTCP15171(S) (102,300±27,840 and 18,440±3,189, respectively; p = 0.0201). Similarly, the lesions of mice experimentally infected with LTCP393(R) contained more CD4^+^ T lymphocytes than those of mice experimentally infected with LTCP15171(S) (1,761,000±257,100 vs. 990,900±124,200; p = 0.0279) (Fig. 3C). No differences were observed in the number of CD8^+^ T lymphocytes or CD4^+^CD25^+^ T cells in the lesions of mice inoculated with LTCP393(R) or LTCP15171(S), and CD4^+^CD25^+^ T cells expressed similar levels of FoxP3, CD103, CTLA-4 and GITR in both groups (Fig. 3E). The numbers of neutrophils, dendritic cells (CD11b^+^CD11c^+^) and macrophages were higher in animals inoculated with LTCP393(R) than in animals inoculated with LTCP15171(S) (p = 0.0086, 0.0339 and 0.0006, respectively); between weeks five and seven, these cell types decreased in number more intensely in lesions from mice inoculated with LTCP15171(S) than in lesions from mice inoculated with LTCP393(R) (Fig. 3C).

Twelve weeks post-infection, the numbers of all cell populations in the animals inoculated with LTCP393(R) remained similar to those observed seven weeks post-infection while the numbers of most cell populations decreased in the animals inoculated with LTCP15171(S). The numbers of B lymphocytes, CD4^+^ and CD8^+^ T lymphocytes, CD4^+^CD25^+^ T cells, and neutrophils were thus significantly higher in animals inoculated with LTCP393(R) than in LTCP15171(S)-inoculated mice. No statistical differences in the numbers of dendritic cells and macrophages were observed between mice inoculated with resistant or susceptible isolates (Fig. 3D). Furthermore, no significant difference was observed in the frequency (%) of FoxP3, CD103, GITR and CTLA-4 positive cells among CD4^+^CD25^+^ cells between mice inoculated with the resistant or susceptible isolates (Fig. 3F).

### Lymph node cells from mice inoculated with LTCP393(R) produce higher levels of IL-4 when challenged with the parasite

**Figure 4:**
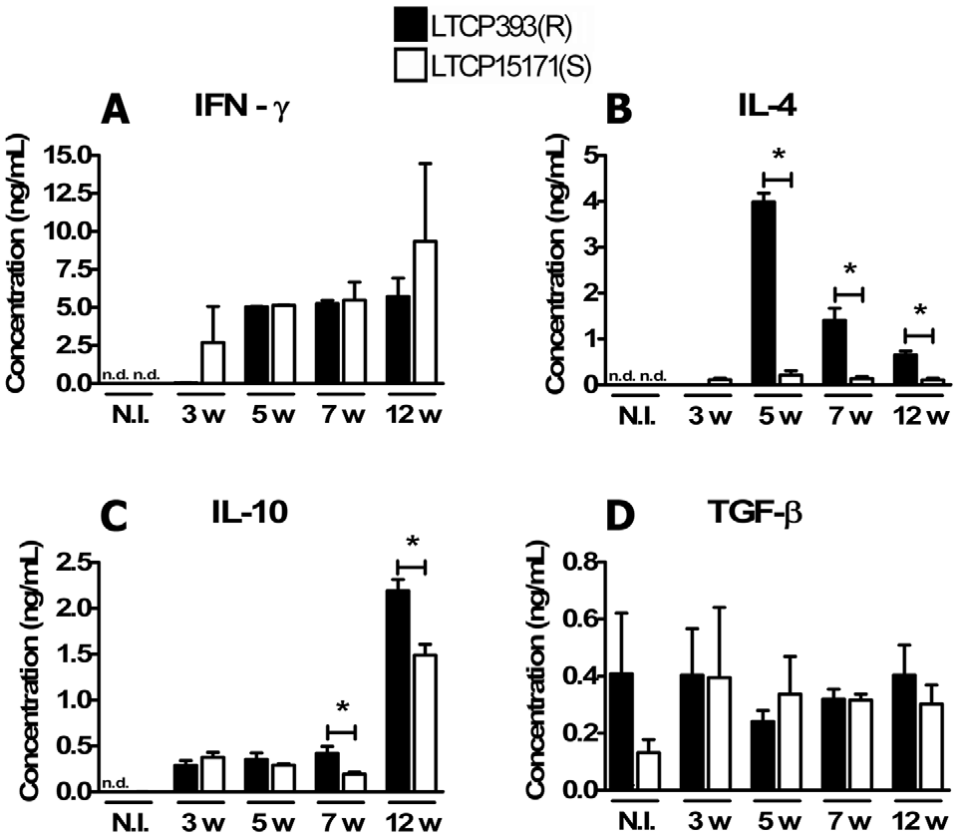
Cytokine production by lymph node cells from LTCP393(R) and LTCP15171(S)-inoculated mice. Cells were isolated from the draining lymph nodes of non infected (N.I.) BALB/c mice or BALB/c mice inoculated with 1×10^6^ stationary phase *L. braziliensis* promastigotes, isolates LTCP393(R) (black bars) or LTCP15171(S) (white bars), at 3, 5, 7 and 12 weeks post infection. 1×10^6^ cells/well were incubated in 48 flat bottom well plates with 500 µl of complete RPMI medium for 72 hours, at 37°C, 5% CO_2_, in the presence of live parasites in a 5∶1 parasite∶cell ratio. The cell cultures supernatants were collected and IFN-γ (A), IL-4 (B), IL-10 (C) and TGF-β (D) productions were quantified by ELISA method. Results are expressed as mean ± standard error of mean, and are representative of 3 independent experiments. n.d. = non-detected. *p<0,05.

We have previously observed that the differences between the disease manifestations in mice inoculated with LTCP393(R) or LTCP15171(S) became evident at later time points of the experimental infection. Therefore, immune responses induced by parasites were examined by measuring the cytokines produced by lymph node cells isolated 3–12 weeks post-infection and stimulated *in vitro* with live stationary phase promastigotes. The average production of IFN-γ was higher in lymph node cells harvested from animals inoculated with LTCP15171(S) three weeks post-infection, than in those from animals inoculated with LTCP393(R); however, this difference was not statistically significant. Lymph node cells harvested from animals inoculated with either isolate 5, 7 or 12 weeks post-infection produced more than 5 ng/ml IFN-γ when stimulated with live parasites. No statistical significant differences were observed between lymph node cells from mice inoculated with resistant or susceptible isolates at these time points (Fig. 4A).

Low levels of IL-4 were produced by lymph node cells isolated from animals inoculated with LTCP15171(S) three weeks post-infection and stimulated with live parasites, but no IL-4 was detected in the lymph node cells from animals inoculated with LTCP393(R) at this time point. In contrast, five weeks post-infection, lymph node cells isolated from animals inoculated with LTCP393(R) and challenged with live parasites produced significantly higher levels of IL-4 (3.987±0.1914 ng/ml) than those isolated from animals inoculated with LTCP15171(S) (0.2122±0.0924 ng/ml; p<0.0001). The production of IL-4 by lymph node cells from mice inoculated with the resistant isolate seven and 12 weeks post-infection was lower than that observed at five weeks post-infection (1.406±0.2616 and 0.6536±0.08217 ng/ml, respectively), but still significantly higher than that observed in animals inoculated with the susceptible parasites (0.135±0.04826 and 0.1071±0.04127 ng/ml; p = 0.0088 and 0.0040, respectively) (Fig. 4B).

Upon stimulation with parasites, IL-10 was detected at low levels in lymph node cells isolated from mice inoculated with either isolate three and five weeks post-infection. Seven weeks after infection, however, IL-10 production increased in cells isolated from mice inoculated with LTCP393(R), and these cells produced higher levels of IL-10 than those isolated from mice inoculated with the susceptible parasite (0.4911±0.07603 and 0.1925±0.02022 ng/ml, respectively; p = 0.028). Twelve weeks post-infection, IL-10 production increased in lymph node cells from mice inoculated with either isolate, but cells from mice inoculated with LTCP393(R) continued to produce higher levels of this cytokine than those from mice inoculated with LTCP15171(S) (2.190±0.1237 ng/ml and 1.488±0.1190 ng/ml, respectively; p = 0.0064) (Fig. 4C). TGF-β production did not significantly differ between stimulated and unstimulated cultures or between cells isolated from mice inoculated with LTCP393(R) and those from mice inoculated with LTCP15171(S) at the time points analyzed. The production of this cytokine throughout the experimental infection remained similar to non-infected mice in all time points analyzed in both groups (Fig. 4D).

### Mice inoculated with LTCP393(R) have increased expression of IL-4 and Arg I in the lesions

**Figure 5:**
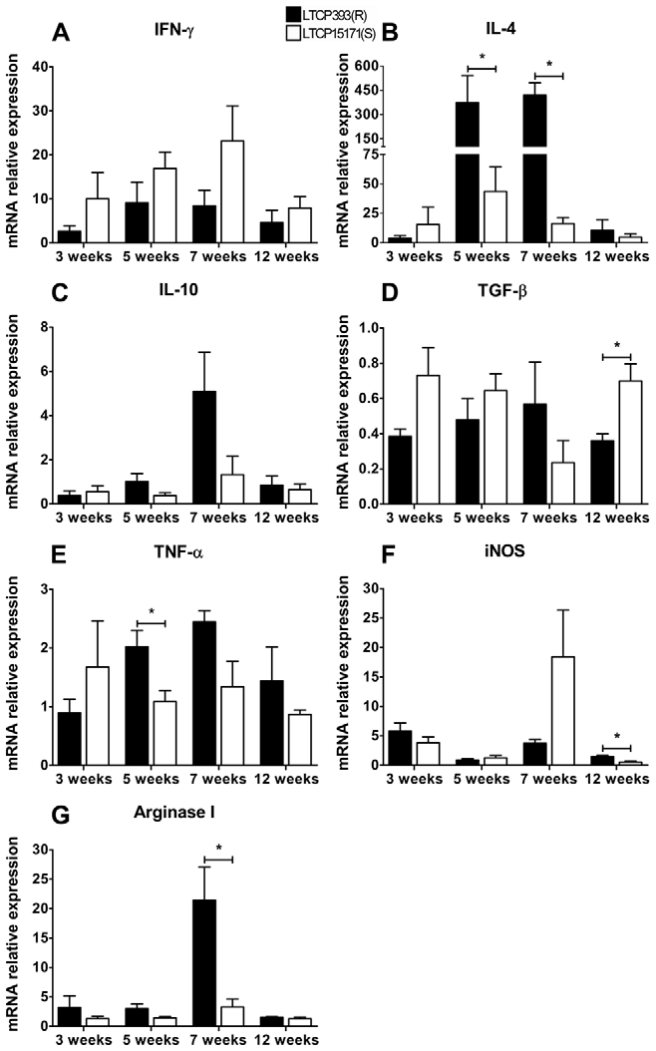
Cytokines and enzymes mRNA expression in the ears from LTCP393(R) and LTCP15171(S)-inoculated mice. The animals were inoculated with 1×10^6^ stationary phase *L. braziliensis* promastigotes, isolates LTCP393(R) (black bars) or LTCP15171(S) (white bars). mRNA was extracted from the ears at 3, 5, 7 and 12 weeks post infection, and the specific mRNAs for IFN-γ (A), IL-4 (B), IL-10 (C), TGF-β (D), TNF-α (E), iNOS (F) and Arg I(G) were detected by real time RT PCR. The specific molecules mRNA expression was normalized based in the endogenous expression of the mRNAm for β-actin. Results are listed as the mean of expression in the ears of infected animals in relation to ears of uninfected ones ± standard error of mean, and are representative of 3 independent experiments. *p<0,05.

The expression patterns of cytokines and the enzymes iNOS and Arg I at the sites of experimental infection, were examined by processing the ears of the experimentally infected mice for mRNA extraction and quantifying mRNA by real time PCR at the same time points at which cytokine production was measured in lymph node cells. Three weeks post-infection, there was no significant difference in the expression of IFN-γ, IL-4, IL-10, TGF-β, TNF-α, iNOS and Arg I mRNA at experimental infection sites between the mice inoculated with the resistant or susceptible parasites (Fig. 5A–G). Similar to our observations in lymph node cultures, five weeks post-infection, the ears of LTCP393(R)-inoculated mice expressed higher levels of IL-4 mRNA than those of LTCP15171(S)-inoculated mice (372.6±168.1 and 43.51±20.81; p = 0.0398) (Fig. 5B), and no significant differences were found in the expression of IFN-γ mRNA (Fig. 5A). No differences were found in IL-10 and TGF-β expression and also in the enzymes iNOS and Arg I expression, at five weeks post infection (Fig. 5C–G). At this same time point, TNF-α was expressed at higher levels in the ears of animals inoculated with LTCP393(R) than those of animals inoculated with LTCP15171(S) (Fig. 5E). Seven weeks post-infection, we observed approximately 20-fold increase in IL-4 mRNA expression in mice inoculated with the resistant isolate compared to that in mice inoculated with the susceptible isolate (419.9±76.33 and 16.23±5.164, respectively; p = 0.0019) (Fig. 5B). Also, Arg I expression was significantly higher in ears of mice experimentally infected with LTCP393(R) compared to mice infected with LTCP15171(S) (21.41±5.615 and 3.282±1.338, respectively; p = 0.02) (Fig. 5G). No statistical difference was detected in IFN-γ and iNOS expression between these groups, although a clear tendency of higher expression of the enzyme was observed in ears of mice infected with the resistant isolate (Fig. 5A and F). Albeit IL-10 mRNA expression increased in the ears of animals infected with the LTCP393(R) isolate at seven weeks post infection, it was not significantly different from that detected in the ears of LTCP15171(S)-inoculated mice. (Fig. 5D). No differences were detected in the expression of TNF-α and TGF-β seven weeks after infection (Fig. 5B and E). Twelve weeks post-infection, the expression levels of IFN-γ, IL-4, IL-10, TNF-α and Arg I were similar in both groups. The only differences observed were in TGF-β and iNOS expression; the cytokine was expressed at higher levels in ears of mice inoculated with LTCP15171(S) while the enzyme was expressed at higher levels in ears of mice inoculated with LTCP393(R) (Fig. 5E and F).

### IL-4 neutralization decreases lesion development and parasite load in animals experimentally infected with LTCP393(R)

**Figure 6:**
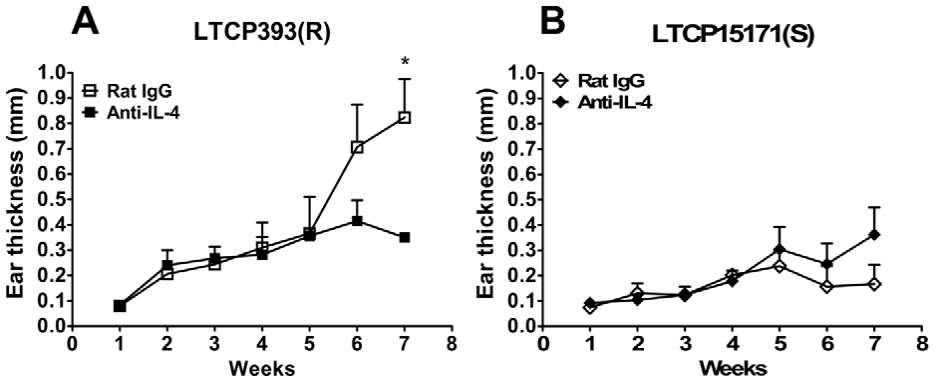
Anti-IL-4 mAb treatment reduces lesion thickness in LTCP393(R)-inoculated BALB/c mice. The animals were inoculated with 1×10^6^ *L. braziliensis* stationary phase promastigotes, isolates (A) LTCP393(R) or (B) LTCP15171(S), in the right ear dermis, treated with anti-IL-4 monoclonal antibody (black signs) or rat IgG (white signs), and the course of lesion development was monitored for 7 weeks. Lesion thickness was determined as the difference between the infected ear and the contralateral one, noninfected. Results are expressed as mean ± standard error of mean, and are representative of 2 independent experiments. *p<0,05.

**Figure 7:**
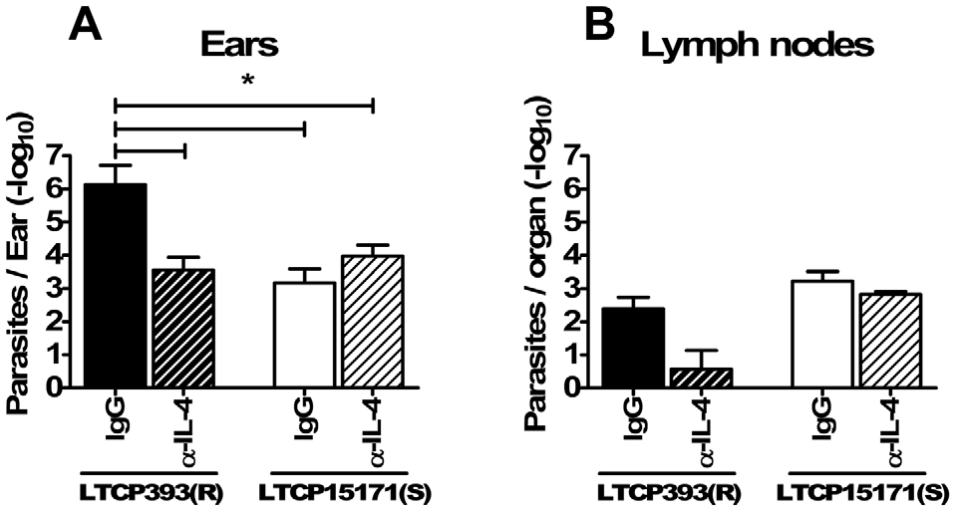
Anti-IL-4 mAb treatment reduces parasite loads in LTCP393(R)-inoculated BALB/c mice lesions. The animals were inoculated at right ear dermis with 1×10^6^ stationary phase promastigotes of *L. braziliensis*, isolates LTCP393(R) (black bars) or LTCP15171(S) (white bars), treated with anti-IL-4 monoclonal antibody (hatched bars) or rat IgG (full bars), and the parasite load estimated at 7 weeks post infection in the ears (A) and draining lymph nodes (B). Results are expressed as mean ± standard error of mean, and are representative of 2 independent experiments. Statistical analysis: ANOVA and Tukey multiple comparision test. *p<0,05.

Because the most striking difference between the two groups was the level of IL-4 production, it was reasonable to associate IL-4 production with the increased susceptibility to experimental infection with the resistant isolate. To test this hypothesis, BALB/c mice were treated with anti-IL-4 mAb before the inoculation with 1×10^6^ stationary phase promastigotes of *L. braziliensis* LTCP393(R) or LTCP15171(S) isolates. The mice were then treated with anti-IL-4 mAb twice a week for seven weeks after infection. During the first six weeks after infection, no differences were detected between mice inoculated with LTCP393(R) and treated with anti-IL-4 mAb and those inoculated with LTCP393(R) and treated with control IgG. Seven weeks after experimental infection, however, lesions in animals treated only with normal IgG increased in thickness while those in animals treated with anti-IL-4 mAb began to decrease significantly in thickness (0.8233±0.151 and 0.35±0.02, respectively; p = 0.036) (Fig. 6A). In mice inoculated with LTCP15171(S), no difference was observed between the control animals and those treated with the anti-IL-4 mAb at any time point following inoculation of parasites (Fig. 6B). Moreover, the depletion of IL-4 in animals inoculated with LTCP393(R) resulted in lesion development similar to that seen in mice inoculated with LTCP15171(S), in which little production of IL-4 was detected. Quantification of the parasite load showed that anti-IL-4 mAb treatment resulted in not only decreased lesion thickness, but also a significant decrease in the parasite loads (∼100×) in the ears of LTCP393(R)-inoculated animals. As observed in the lesion measurements, parasite load in anti-IL-4 treated LTCP393(R)-inoculated mice ears was similar to that observed in LTCP15171(S)-inoculated mice (Fig. 7A). Although the average of parasite loads in lymph nodes of anti-IL-4 mAb treated LTCP393(R)-inoculated mice were lower compared to rat IgG treated counterparts, no significant difference was observed (Fig. 7B). In mice inoculated with LTCP15171(S), no difference in lymph node parasite loads were observed between anti-IL-4 mAb and rat IgG treated animals (Fig. 7B).

## Discussion

In this work, we describe an experimental model for the evaluation of the immunomodulatory effects of different isolates of *L. braziliensis* that result in distinct disease manifestations. Using this model, we show that immunomodulation by IL-4 contributes to the different disease outcomes observed upon infection with different *L. braziliensis* isolates.

Upon inoculation of BALB/c mice with *L. braziliensis* isolates LTCP393(R) and LTCP15171(S) we observed clearly two distinct patterns of disease development in the mice. The increased severity of the disease caused in mice by the resistant parasites inoculation was due to both, higher inflammation and increased parasite burdens that could be observed only at later times post-infection.

In models of *L. major* infection, it is clearly established that the type of inflammatory infiltrate may interfere with the infection outcome. CD4^+^ and CD8^+^ T cells are important in inducing protection via Th1 responses [5]–[9], [37], while neutrophils may act as a vehicle for silent entry of *L. major* into macrophages, favoring parasite survival [38], [39]. However, due to few studies concerning about this issue, the importance of each cellular population in *L. braziliensis* infection remains unknown.

Therefore, we analyzed the possible roles of cell populations in inflammatory infiltrate for *L.braziliensis* disease outcomes, via inoculation of LTCP393(R) or LTCP15171(S) *L. braziliensis* isolates. Even presenting different disease manifestations, in both groups, the inflammatory infiltrates were composed mainly by CD4^+^ T lymphocytes and neutrophils. We found differences in the number of cells in some infection time points, however, the frequency of each cell population thus varied following the same pattern in both groups. We observed that all the cell populations analyzed increased or decreased following the same pattern in both groups, according to parasite load. Additionally, no specific cell population was altered differentially between the groups throughout infection, showing no contribution of any specific cell population in the disease outcomes observed in our model. However, our results provide the first evidence that CD4^+^CD25^+^FoxP3^+^ regulatory T cells (Treg), which have the capacity to suppress Th1 effector responses and favor *Leishmania* survival [40]–[45], are recruited to the infection sites in *L. braziliensis*-infected mice.

The differences in the disease outcomes in our experimental model could be due to differences in the induction of specific T cell responses to the parasites of the distinct isolates. For this purpose, we analyzed the production of Th1 cytokines, related to protection to cutaneous leishmaniasis [7], [9], [16], [17], [46]–[49], and Th2 and Treg cytokines, related to susceptibility in murine models of cutaneous leishmaniasis [50], [11], [13], the two last of which have their role poorly characterized in *L. braziliensis* infection.

In our model, the difference in disease outcomes was not due to differential induction of Th1 responses by the isolates, since IFN-γ production in both groups was similar throughout the infection. Also, IL-10 and TGF-β were expressed similarly in the lesions throughout the critical points of infection, thus discarding the possibility of any strong interference of differential Treg activity in the lesion site could be determinant for the disease outcomes. Therefore, during the later phases of infection, IL-10 production increased in lymph node cultures from mice infected with both isolates, mainly in LTCP393(R)-inoculated animals. Since this increase occurred only when the lesions were healing or healed, we believe that it may reflect the homeostatic control of the pro-inflammatory responses, especially in LTCP393(R)-inoculated mice, that presented a more robust inflammatory response at this time point. On the other hand, increased IL-10 production may also be responsible for the increase in the number of parasites in the lymph nodes during the later phases of infection. Moura et al. [32] also demonstrated an increase in the number of parasites in lymph nodes of *L. braziliensis*-infected BALB/c mice in which lesions were cured. It has been shown that IL-10 suppresses effector responses, preventing sterile cure which confers immunity to re-infection with *L. major* [12]. The ability of IL-10 to control parasite numbers may also be important in maintaining immunity to *L. braziliensis*; however, it could also represent an evolutionary success of *L. braziliensis* parasites in maintaining a transmissible reservoir in the host without causing disease.

TNF-α expression was also characterized, but this is a cytokine that suffers posttranscriptional regulation, through binding of proteins to a region called adenine urenine rich element (ARE), present in TNF-α mRNA transcripts [51], and a better characterization is needed to establish its role. However, in our case, although TNF-α is related to protection against infection [9], [48], [16], our finding of increased TNF-α mRNA expression in lesions of LTCP393(R)-inoculated mice five weeks post-infection, may be associated to Th2 responses. In the skin, keratinocytes may produce TSLP, that up-regulates OX40L expression in dendritic cells, which in turn drives differentiation of Th2 cells that produce IL-4, IL-5, IL-13 and TNF-α [52]–[54]. The role of cytokines produced by keratinocytes in *L. braziliensis* infection is an important issue that must be investigated in future works.

In fact, the most striking difference we found between mice infected with the resistant or susceptible isolates was in Th2 responses. IL-4, lesion size and parasite load dramatically increased concomitantly in LTCP393(R)-inoculated mice compared to LTCP15171(S)-inoculated animals. Also, IL-4 neutralization dropped lesion size and parasite loads in lesions of LTCP393(R)-inoculated mice to the same levels found in LTCP15171(S)-inoculated animals, while had no effect in the last ones. This showed that induction of IL-4 production by LTCP393(R) was responsible for the differences in lesion development, susceptibility to parasite replication and disease persistence between mice infected with the different isolates.

It would be expected that IL-4 neutralization led to higher IFN-γ production, increasing parasite killing. Therefore, we did not observe any increase in IFN-γ production upon IL-4 neutralization (data not shown). In *L. major* infection, different grades of resistance to infection could be seen in mice that produce similar levels of IFN-γ but varying levels of IL-4, suggesting that the magnitude of the IL-4 response determines the severity of disease more than IFN-γ [55]. Also in patients infected with *L. braziliensis*, the concomitant production of IL-4 together with IFN-γ is observed, and it has been proposed that it favors parasite survival and impairs spontaneous lesion healing. [56]. Likewise, even though both isolates induce similar levels of IFN-γ production, experimental infection with the LTCP393(R) *L. braziliensis* isolate induces production of higher levels of IL-4, which favors the survival of this parasite and increases disease severity.

IL-4 is a potent activator of the enzyme arginase I (Arg I), which competes with iNOS for L-arginine that is used for the synthesis of NO, in macrophages. By consuming L-arginine, Arg I decreases the availability of this substrate for iNOS, resulting in diminished production of NO, and inhibition of inflammation [57]. Therefore, there is not a reciprocal inhibition between the enzymes, and iNOS and Arg I can be concomitantly expressed in macrophages at the mRNA and protein levels upon stimulation [58], [59]. Especially in a microenvironment where Th1 and Th2 responses are taking place at the same time, that is the case in LTCP393(R)-inoculated mice, this feature occurs, as we showed. Despite expressing the same levels of iNOS than LTCP15171(S)-inoculated mice, the increased Arg I expression in LTCP393(R)-inoculated mice suggests that increased IL-4 production in these animals leads to increases in Arg I in the macrophages, resulting in decreased production of NO, favoring parasite survival, which results in a more severe disease. In lymph nodes, however, IL-4 seems not to interfere in the parasite elimination as in the lesions, since high levels of IL-4 in LTCP393(R)-infected mice did not result in increased parasite load (Fig. 2). Similarly, IL-4 depletion did not result in significant parasite reduction in the organ. These data show that the control of parasite growth and/or parasite elimination in the periphery is distinct than that of lymphoid organs. We do not know the mechanisms by which this feature occurs, although it is possible that IL-10 could be involved. Also, APCs as dendritic cells and macrophages, are able to kill the parasites in the dependence of the microenvironment [60], [61]. In peripheral sites, these cells act mainly as “killer cells”, while, upon migration to lymphoid organs, they change their profile to “antigen presenting cells” [62]–[64].

In conclusion, our model allowed us to suggest that in addition to intrinsic resistance to drugs and NO, the immunomodulation towards Th2 response induced by certain parasite strains may account for more severe lesions, increased parasite burdens, and increased length of disease. We may ultimately propose that even in the presence of IFN-γ, IL-4 production increases arginase I expression and impairs the parasite killing in lesion site. This characteristic of some parasite isolates can be considered a susceptibility factor for *L. braziliensis* infection. The models of infection described here will be useful in the identification of immunological targets to control *L. braziliensis* infection.
